# Effects of acute and chronic administration of trace amine-associated receptor 1 (TAAR1) ligands on in vivo excitability of central monoamine-secreting neurons in rats

**DOI:** 10.1038/s41380-022-01739-9

**Published:** 2022-09-01

**Authors:** Daniil Grinchii, Marius C. Hoener, Talah Khoury, Roman Dekhtiarenko, Reyhaneh Nejati Bervanlou, Daniela Jezova, Eliyahu Dremencov

**Affiliations:** 1 Institute of Molecular Physiology and Genetics, Centre of Biosciences, Slovak Academy of Sciences, Bratislava, Slovakia; 2 Roche Innovation Center Basel, F. Hoffmann-La Roche Ltd, Basel, Switzerland; 3 Institute of Experimental Endocrinology, Biomedical Research Center, Slovak Academy of Sciences, Bratislava, Slovakia

**Keywords:** Predictive markers, Neuroscience, Physiology

## Abstract

Trace amine-associated receptor 1 (TAAR1) has been recently identified as a target for the future antidepressant, antipsychotic, and anti-addiction drugs. Full (e.g. RO5256390) and partial (e.g. RO5263397) TAAR1 agonists showed antidepressant-, antipsychotic- and anti-addiction-like behavioral effects in rodents and primates. Acute RO5256390 suppressed, and RO5263397 stimulated serotonin (5-HT) neurons of the dorsal raphe nucleus (DRN) and dopamine neurons of the ventral tegmental area (VTA) in brain slices, suggesting that the behavioral effects of TAAR1 ligands involve 5-HT and dopamine. For more comprehensive testing of this hypothesis, we examined acute and chronic effects of RO5256390 and RO5263397 on monoamine neurons in in vivo conditions. Excitability of 5-HT neurons of the DRN, noradrenaline neurons of the locus coeruleus (LC), and dopamine neurons of the VTA was assessed using single-unit electrophysiology in anesthetized rats. For acute experiments, RO5256390 and RO5263397 were administered intravenously; neuronal excitability after RO5256390 and RO5263397 administration was compared to the basal activity of the same neuron. For chronic experiments, RO5256390 was administered orally for fourteen days prior to electrophysiological assessments. The neuronal excitability in RO5256390-treated rats was compared to vehicle-treated controls. We found that acute RO5256390 inhibited 5-HT and dopamine neurons. This effect of RO5256390 was reversed by the subsequent and prevented by the earlier administration of RO5263397. Acute RO5256390 and RO5263397 did not alter the excitability of LC noradrenaline neurons in a statistically significant way. Chronic RO5256390 increased excitability of 5-HT neurons of the DRN and dopamine neurons of the VTA. In conclusion, the putative antidepressant and antipsychotic effects of TAAR1 ligands might be mediated, at least in part, via the modulation of excitability of central 5-HT and dopamine neurons.

## Introduction

Trace amines are endogenous molecules that are present in the mammal brain in low concentrations. The trace amines include phenethylamines (phenethylamine, n-methylphenethylamine, phenylethanolamine), m- and p-tyramine, 3-methoxytyramine, n-methyltyramine, m- and p-octopamine, synephrine, and tryptamine. Trace amines are closely related to the “classical” monoamines from the structural and metabolic points of view [1].

The discovery of trace amine-associated receptors (TAARs) in 2001, which are expressed in key brain areas, where the modulation of dopaminergic and serotonergic neurotransmission occurs, suggested that trace amines, despite their low concentrations, may perform important signaling functions in the central nervous system [2, 3]. There are nine TAARs in human, all GPCRs, TAAR1, TAAR2, TAAR5, TAAR6, TAAR8 and TAAR9 are functional. TAAR1 is G_S_-, TAAR5 G_S_- and/or G_Q_-, and TAAR8 is G_I_-coupled [4]. TAAR1 receptors are widely expressed in the dorsal raphe nucleus (DRN), ventral tegmental area (VTA), entorhinal and prefrontal cortex, hippocampus, and hypothalamus [5]. Unlike “classical” receptors to monoamines, which are primarily expressed on the cell membrane, TAAR1 receptors are predominantly expressed on the intracellular compartments of the neurons [6].

TAAR1 receptors are of special interest as a target for the future antidepressant drugs. Three lines of evidence support this hypothesis. First, TAAR1 ligands demonstrated antidepressant-like effects in rodents and in primates. The partial agonist RO5263397 decreased immobility time of rats undergoing the forced swim test (FST), and both RO5263397 and the full TAAR1 agonist RO5256390 improved differential reinforcement for low-rate (DRL) scores in Marmoset monkeys [7]. Second, TAAR1 receptors are densely expressed in brain areas associated with depression and antidepressant drugs response, such as dorsal raphe nucleus (DRN) and ventral tegmental area (VTA) [8]. Third, TAAR1 ligands were shown to modulate monoamine neurotransmission. RO5256390 [7] and another agonist of TAAR1, RO5166017 [9] were shown to inhibit ex vivo excitability of 5-HT and dopamine neurons in brain slices. Interestingly, transgenic mice overexpressing TAAR1 showed increased ex vivo excitability of 5-HT and dopamine neurons [8]. The same study showed, using in vivo microdialysis, that TAAR1-overexpressing mice have increased concentrations of dopamine in the nucleus accumbens (NAcc) and 5-HT levels in the medial prefrontal cortex (PFC). Finally, De Gregorio and co-authors reported a stimulatory effect of the TAAR1 receptor antagonist EPPTB on VTA dopamine neurons in in vivo conditions. They provide evidence that pre-treatment with EPPTB blocked d-lysergic acid diethylamide (LSD)-induced inhibition of VTA dopamine neurons [10].

Although the effects of acute administration of TAAR ligands on the excitability of monoamine-secreting neurons were previously studied in ex vivo conditions, and the effect of EPPTB on dopamine neurons was examined in in vivo conditions, neither acute, nor chronic effects of TAAR1 ligands on in vivo excitability of 5-HT or noradrenaline secreting neurons were previously investigated. To the authors’ best knowledge, chronic TAAR1 ligands effect on dopamine neuronal firing activity has not yet been assessed as well. The aim of this study was to assess the effects of RO5256390 and RO5263397 on in vivo excitability of rat 5-HT, noradrenaline, and dopamine-secreting neurons. In order to investigate the role of extracellular 5-HT in the RO5256390-mediated modulation of activity of the 5-HT neurons, we also studied the effects of acute RO5256390 on 5-HT neuronal firing activity after the inhibition of 5-HT synthesis by p-chlorophenylalanine (PCPA).

## Methods

### Animals

Adult male Wistar rats (250–300 g) were ordered from the Animal Breeding facility of the Institute of Experimental Pharmacology and Toxicology, Centre for Experimental Medicine, Slovak Academy of Sciences (Dobra Voda, Slovakia). Animals were housed under standard laboratory conditions (temperature: 22 ± 2 °C, humidity: 55 ± 10%) with a 12 h light/12 h dark cycle (lights on at 7 a.m.). Pelleted food and tap water were available ad libitum. All experimental procedures were approved by the Animal Health and Animal Welfare Division of the State Veterinary and Food Administration of the Slovak Republic (Permit number Ro 3592/15-221) and confirmed to the Directive 2010/63/EU of the European Parliament and of the Council on the Protection of Animals Used for Scientific Purposes.

### Chemicals

RO5256390 and RO5263397 were received as a gift from Roche Innovation Center Basel (Basel, Switzerland). RO5256390 was dissolved in 0.3% polysorbate-80 in distilled water (for the oral administration) or in 0.3% polysorbate-80 in 0.9% sodium chloride (NaCl; saline) solution in distilled water (for the intravenous: i.v. administration). RO5263397 was dissolved in 0.3% polysorbate-80 in saline solution. PCPA was dissolved in 20% solution of (2-hydrosypropil)-β-cyclodextrin. All other drugs chemicals were ordered from Lambda Life s.r.o. (Bratislava, Slovakia) and dissolved in saline.

### Inhibition of 5-HT synthesis

Inhibition of 5-HT synthesis was induced by intraperitoneal administration of PCPA. PCPA was injected at the dose of 300 mg/kg/day for 3 days, as described previously [11]. To investigate the effect of RO5256390 on 5-HT neuronal firing under conditions of reduced 5-HT synthesis, this compound was administered 24 h after the last PCPA injection.

### Acute RO5256390 and RO5263397 treatment

In experiments aiming to test the effect of acute RO5256390 and RO5263397 on the excitability of 5-HT and dopamine neurons, RO5256390, RO5263397, or vehicle were administered, after a neuron was identified and its basal firing activity was recorded for 2 min, via a catheter placed in a femoral vein. In the first series of experiments, RO5256390 was administered at cumulative doses of 50–1000 μg/kg (i.v.). After the last RO5256390 exposure, RO5256397 was administered at cumulative doses of 50–1000 μg/kg (i.v.). After the last RO5256390 administration, 8-OH-DPAT (for 5-HT neurons) or haloperidol (for dopamine neurons) were administered, each at the dose of 0.1 mg/kg (i.v.). In second series of experiment, RO5256397 was administered firstly, at cumulative doses of 50–1000 μg/kg (i.v.). After the last RO5256397 administration, RO5256390 was administered at cumulative doses of 50–1000 μg/kg (i.v.). After the last RO5256390 administration, WAY100135 (for 5-HT neurons) or apomorphine (for dopamine neurons) were administered, each at the dose of 0.1 mg/kg (i.v.).

### Chronic RO5256390 treatment

In experiments aiming to test the effect of chronic RO5256390 on the excitability of 5-HT and dopamine neurons, rats were randomly divided into vehicle and RO5256390 groups. Animals were pre-treated with RO5256390 (orally, 1.5 mg/kg, twice a day at 09:00 and 17:00) or its vehicle for fourteen consecutive days preceding the day of electrophysiological assessments. We administered twice daily because of its relatively short biological half-time (~7 h) [12]. The last vehicle or RO5256390 injection was performed on day 15th, and the electrophysiological assessments were performed one hour thereafter.

### Electrophysiology in vivo

In vivo electrophysiological experiments were performed as previously described [13–19]. Animals were anesthetized by chloral hydrate (400 mg/kg, i.p.) and mounted in the stereotaxic frame (David Kopf Instruments, Tujunga, CA). Body temperature was maintained between 36 and 37 °C with a heating pad (Gaymor Instruments, Orchard Park, NY, USA). The scalp was opened, and a 3 mm hole was drilled in the skull for insertion of electrodes. Glass-pipettes were pulled with a DMZ-Universal Puller (Zeitz-Instruments GmbH, Martinsried, Germany) to a fine tip approximately 1 μm in diameter and filled with 2 M NaCl solution. Electrode impedance ranged from 4 to 6 MΩ. The pipettes were inserted into the DRN (7.8–8.3 mm posterior to bregma and 4.5–7.0 mm ventral to brain surface), LC (8.0–8.3 mm posterior to bregma, 1.2–1.4 mm lateral to the midline, and 5.5–7.5 mm ventral to the brain surface), or VTA (4.5–5.5 mm posterior to bregma, 0.6–0.8 mm lateral to the midline, and 7.0–8.5 mm ventral to the brain surface) [20] by hydraulic micro-positioner (David Kopf Instruments, Tujunga, CA). The action potentials generated by monoamine-secreting neurons were recorded using the AD Instruments Extracellular Recording System (Dunedin, New Zealand).

The 5-HT neurons were identified by bi- or tri-phasic action potentials with a rising phase of long duration (0.8–1.2 ms) and regular firing rate of 0.5–5.0 Hz [15, 21]. Noradrenaline LC neurons were recognized by action potentials with a long-duration rising phase (0.8–1.2 ms), regular firing rate of 0.5–5.0 Hz, and a characteristic burst discharge in response to nociceptive pinch of the contralateral hind paw [21]. Dopamine neurons were recognized by tri-phasic action potentials lasting between 3 and 5 ms with a rising phase lasting over 1.1 ms, inflection or “notch” during the rising phase, marked negative deflection, irregular firing rate of 0.5–10 Hz, mixed single-spike and burst firing with characteristic decrease of the action potentials amplitude within the bursts [22].

The same number of electrode descents per brain structure (four for the DRN and five for the VTA) were made in vehicle- and RO5256390-pre-treated rats. All spontaneously active neurons were recorded for two minutes. The firing characteristics of the neurons in RO5256390-administered rats were compared to these in vehicle-administered controls.

After completion of electrophysiological recordings, the animals were euthanized by overdose of chloral hydrate. In selected animals, the electrode tip location was labeled by electrolytic lesion using a direct current (DC) of 0.5 mA for 15 s, as previously described [16, 17]. The brains were removed and fixed in 10% paraformaldehyde for 24 h, and afterward in 30% sucrose for 7 days. Frozen sections were cut at 50 mm and examined under a light microscope to verify the placement of the electrode tip in the DRN, LC, or VTA (see supplementary materials, Fig S1).

### Data analysis

Action potentials (spikes) of 5-HT, noradrenaline, and dopamine neurons were detected using the spike sorting algorithm, with the version 6.02 of Spike2 software (Cambridge Electronic Design, Cambridge, UK). The neuronal firing rate and burst activity characteristics were calculated using the burstiDAtor software (www.github.com/nno/burstidator). The onset of a burst was signified by the occurrence of two spikes with ISI < 0.08 s for noradrenaline and dopamine neurons, and ISI < 0.01 s for 5-HT neurons. The termination of a burst was defined as an ISI > 0.16 s for noradrenaline and dopamine neurons [23, 24] and ISI > 0.010 s for 5-HT neurons [25]. Statistical assessments were performed using SigmaPlot 12.5 software (Systat Software Inc, Chicago, IL, USA). Analysis of variance (ANOVA) for repeated measures, followed by Bonferroni post-hoc test, was used to determine the effect of acute RO5256390 and RO5263397 on the spontaneous firing activity of 5-HT, noradrenaline, and dopamine neurons. Two-tailed Student’s t-test was used to determine the effect of chronic RO5256390 on the excitability of 5-HT and dopamine neurons. The probability of *p* ≤ 0.05 was considered significant. Statistical tests were preceded by Shapiro–Wilk normality test and equal variance test.

## Results

### Excitability characteristics of monoamine neurons

The firing rates of monoamine neurons, as well as the other characteristics of their excitability, such as the density of the spontaneously active neurons, mean ISI, and parameters of their bursting activity (frequency of the bursts, percent of spikes occurring within the bursts and mean number of spikes per burst) are provided in Table 1.

**Table 1:** Characteristics of excitability of 5-HT neurons of the dorsal raphe nucleus (DRN) and dopamine neurons of the ventral tegmental area (VTA) in control animals.

Characteristic	Control	Chronic RO5256390
A: Serotonin (5-HT) neurons of the dorsal raphe nucleus (DRN)		
Number of spontaneously active neurons per electrode descent	3.75 ± 0.70	3.79 ± 0.67
Percent of neurons with burst firing	62 ± 12	74 ± 3
Frequency of the action potentials, Hz	2.26 ± 0.21	2.39 ± 0.17
Frequency of the bursts, Hz	0.12 ± 0.02	0.19 ± 0.02*
Percent of the action potentials occurring in bursts	10 ± 2	15 ± 2
Mean number of the action potentials in burst	2.18 ± 0.07	2.17 ± 0.05
Mean interspike interval, ms	13.91 ± 0 .33	12.78 ± 0.31*
B: Dopamine neurons of the ventral tegmental area (VTA)		
Number of spontaneously active neurons per electrode descent	4.67 ± 0.81	5.81 ± 0.70
Percent of neurons with burst firing	100	100
Frequency of the action potentials, Hz	3.89 ± 0.28	6.85 ± 0.43***
Frequency of the bursts, Hz	0.44 ± 0.04	0.63 ± 0.04***
Percent of the action potentials occurring in bursts	46 ± 3	66 ± 2***
Mean number of the action potentials in burst	4.60 ± 0.40	8.63 ± 0.8***
Mean interspike interval, ms	51.18 ± 2.24	53.44 ± 1.56

All dopamine neurons of the VTA exhibit burst firing according to their identification criteria. *p < 0.05 and ***p < 0.001, two-tailed Student's t-test.

### Acute RO5256390 inhibits 5-HT neurons; RO5256397 prevents and reverses RO5256390-induced inhibition of 5-HT neuronal firing activity

**Fig. 1:**
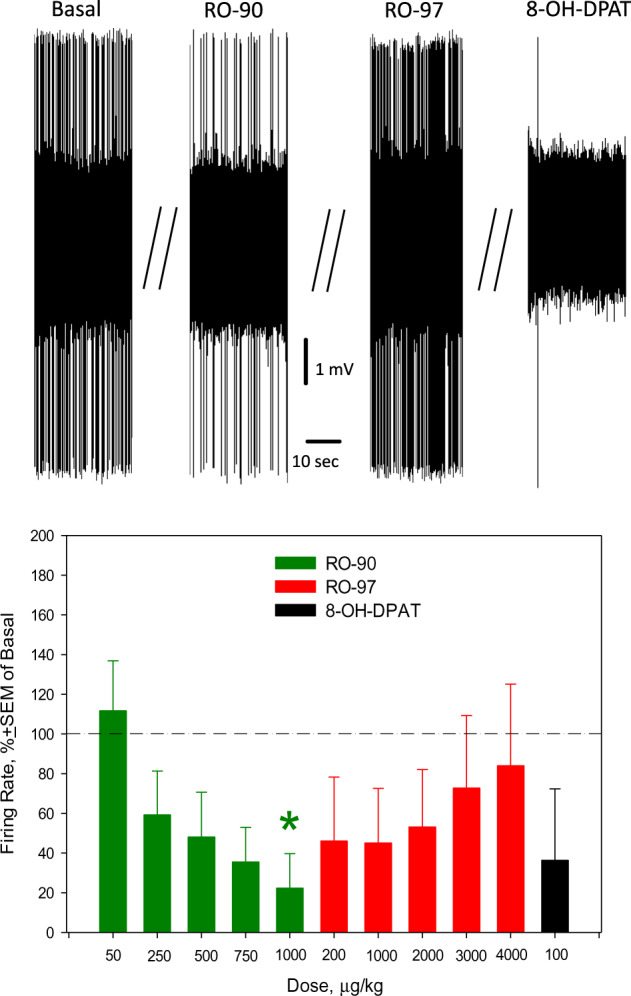
Effects of acute consecutive administration of RO5256390 (RO-90), RO5263397 (RO-97), and 8-OH-DPAT on the excitability of serotonin (5-HT) neurons of the dorsal raphe nucleus (DRN). Up: representative recording from a 5-HT neuron; bottom: summary effect from 7 neurons from 7 rats; *p < 0.05, Bonferroni post-hoc test.

**Fig. 2:**
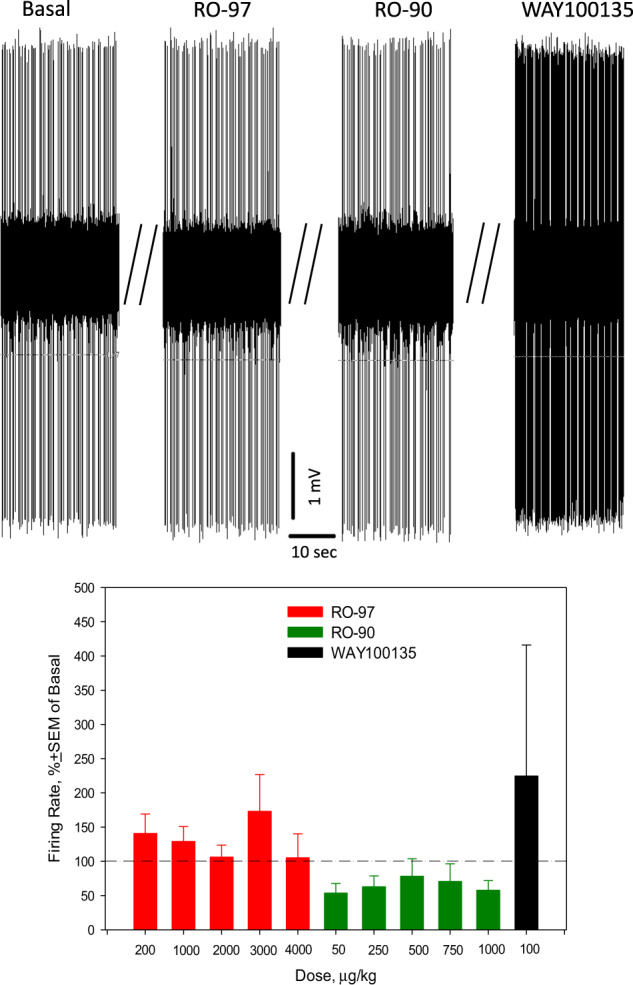
Effects of acute consecutive administration of RO5263397 (RO-97), RO5256390 (RO-90), and WAY100135 on the excitability of serotonin (5-HT) neurons of the dorsal raphe nucleus (DRN). Up: representative recording from a 5-HT neuron; bottom: summary effect from 9 neurons from 9 rats.

When acute RO5256390 was administered first, it dose-dependently altered the firing activity of 5-HT neurons of the DRN. Subsequent administration of RO5263397 reversed the RO5256390-induced inhibition of 5-HT neuronal firing activity, in a dose-dependent manner. Finally, 8-OH-DPAT re-inhibited the excitability of 5-HT neurons (Fig. 1). ANOVA for repeated measures confirmed the statistical significance of the effect of RO5256390, RO5263397, and 8-OH-DPAT on the excitability of 5-HT neurons (*F*_df11,57_ = 2.41, *p* < 0.05; data from 7 neurons from 7 rats). When RO5263397 was administered first, it did not alter the excitability of 5-HT neurons. Subsequent administration of RO5256390 tended to inhibit the 5-HT neuronal activity and the final injection of WAY100135 reversed this inhibition, however, these effects were not statistically significant (data from 9 neurons from 9 rats, Fig. 2). Acute administration of RO5263397 led to a significant decrease in the burst activity of 5-HT neurons (frequency of bursts: *F*_1,9_ = 33.74, *p* = 0.004; percent of spikes occurring in the bursts: *F*_1,9_ = 9.47, *p* = 0.04; supplementary materials, Tab. S1) while acute RO5256390 tended to induce an increase. Administration of vehicle did not alter the excitability of 5-HT neurons (data from 5 neurons from 5 rats, supplementary materials, Fig. S2). In PCPA-pre-treated animals, RO5256390 did not alter the excitability of 5-HT neurons of the DRN in a statistically significant way (data from 5 neurons from 4 rats, supplementary materials, Fig. S3).

### Acute RO5256390 and RO5256397 do not alter the excitability of noradrenaline neurons

Acute RO5256390 and RO5256397 did not alter the excitability of noradrenaline neurons of the LC in a statistically significant way, regardless the order of their administration (RO5256390 administered first: data from 4 neurons from 4 rats; RO5256397 administered first: data from 6 neurons from 6 rats; supplementary materials, Fig. S4). The burst firing of noradrenaline neurons of the LC was not affected by acute RO5256390 or RO5256397 administration (Supplementary materials, Table S1).

### Acute RO5256390 inhibits dopamine neurons; RO5256397 prevents and reverses RO5256390-induced inhibition of dopamine neuronal firing activity

**Fig. 3:**
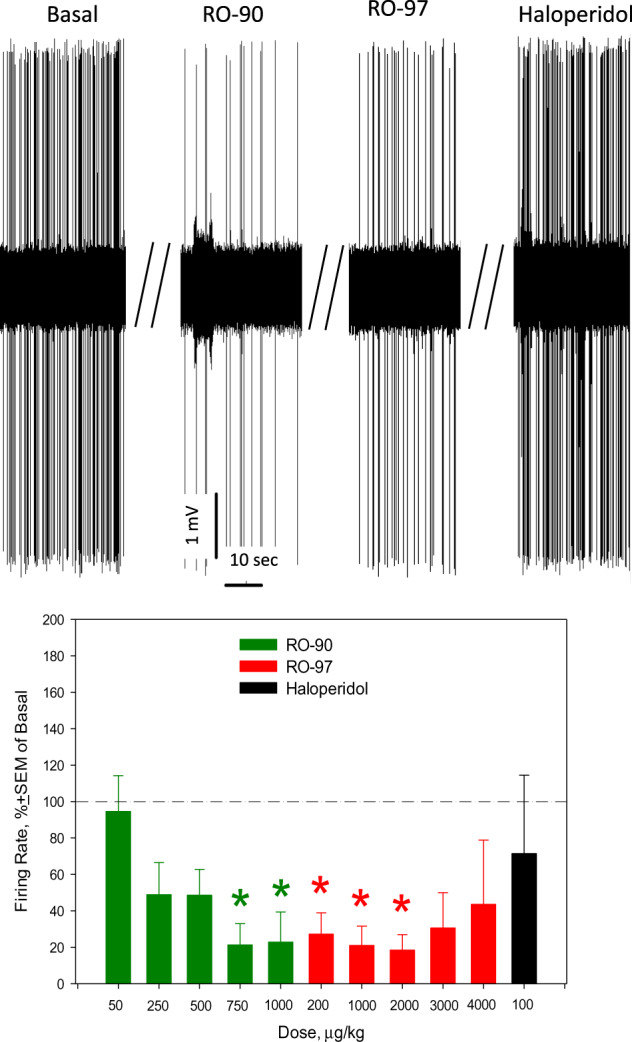
Effects of acute consecutive administration of RO5256390 (RO-90), RO5263397 (RO-97), and haloperidol on the excitability of dopamine neurons of the ventral tegmental area (VTA). Up: representative recording from a 5-HT neuron; bottom: summary effect from 10 neurons from 10 rats; *p < 0.05, Bonferroni post-hoc test.

**Fig. 4:**
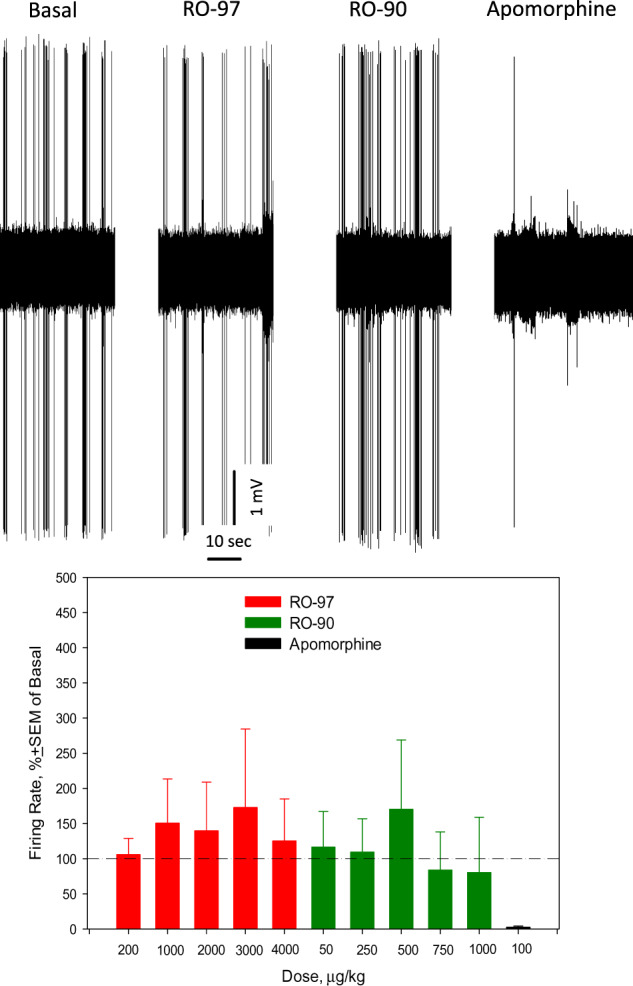
Effects of acute consecutive administration of RO5263397 (RO-97), RO5256390 (RO-90), and apomorphine on the excitability of dopamine neurons of the ventral tegmental area (VTA). Up: representative recording from a 5-HT neuron; bottom: summary effect from 10 neurons from 10 rats.

When RO5256390 was administered first, it dose-dependently altered the firing activity of dopamine neurons of the VTA. Subsequent administration of RO5263397 partially reversed the RO5256390-induced inhibition of dopamine neuronal firing activity, in a dose-dependent manner. The complete recovery of dopamine neuronal firing activity was observed after the subsequent injection of haloperidol (Fig. 3). ANOVA for repeated measures confirmed the statistical significance of the effect of RO5256390, RO5263397, and haloperidol on the excitability of dopamine neurons (*F*_df11,119_ = 2.82, *p* < 0.01, data from 10 neurons from 10 rats). When RO5263397 was administered first, it did not alter the excitability of dopamine neurons. Neither did RO5256390 that was injected subsequently. The final administration of apomorphine suppressed the firing rate of dopamine neurons, however, the statistical effects of RO5263397, RO5256390, and apomorphine were not statistically significant (data from 10 neurons from 10 rats, Fig. 4). With regards to the burst activity of dopamine neurons, acute RO5256390 decreased the frequency of bursts (*F*_1,19_ = 16.28, *p* = 0.003). Acute RO5263397 did not alter the burst firing of dopamine neurons of the VTA (supplementary materials, Tab. S1). Administration of vehicle did not alter the excitability of dopamine neurons (data from 5 neurons from 5 rats, Supplementary materials, Fig. S2).

### Chronic RO5256390 does not alter the firing rate but stimulates the burst mode of firing of 5-HT neurons

**Fig. 5:**
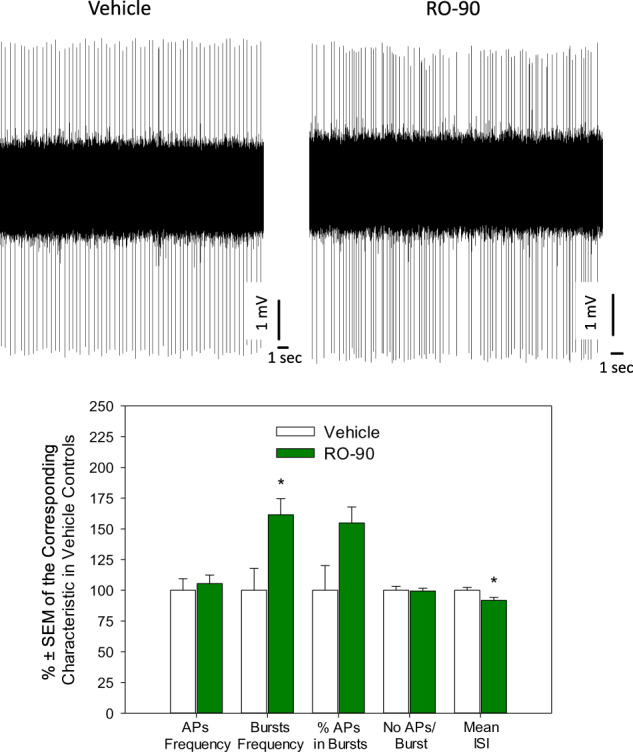
Effect of chronic administration of RO5256390 (RO-90) on the excitability of 5-HT neurons of the ventral tegmental area (VTA). Up: representative recordings from 5-HT neurons from a vehicle- (left) and RO5256390-treated rat (right); bottom: summary effect from 61 neurons from 5 vehicle- and 72 neurons from 5 RO5256390-treated rats; AP action potential (spike); ISI interspike interval; *p < 0.05, two-tailed Student’s *t*-test.

Chronic RO5256390 led to a significant (*p* < 0.05, two-tailed Student’s *t*-test, data from 61 neurons from 5 vehicle- and 72 neurons from 5 RO5256390-treated rats) increase in the frequency of burst-like firing of 5-HT neurons and tended to increase the percent of spikes which occurred within the burst (*p* = 0.07, two-tailed Student’s *t*-test). As a result, the mean ISI in RO5256390-treated rats was significantly shorter than in controls (p < 0.05). Other characteristics of excitability of 5-HT neurons were not affected by RO5256390 (Fig. 5; the characteristics of the neuronal excitability in RO5256390-treated rats are shown as %±SEM of the corresponding characteristics in the vehicle-treated controls to allow the presentation of these characteristics at the same graph, statistical assessment was performed on the actual, non-normalized values).

### Chronic RO5256390 boosts the firing rate and burst activity of dopamine neurons

**Fig. 6:**
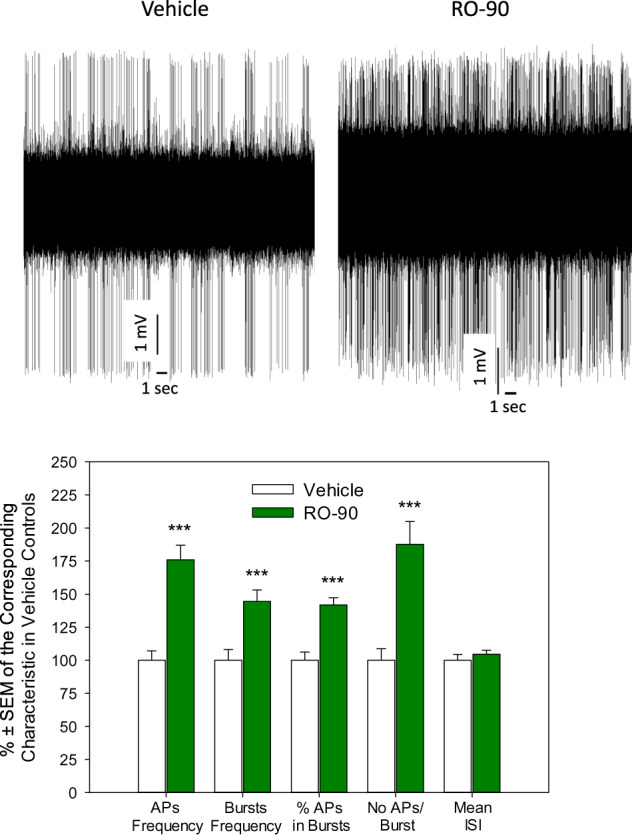
Effect of chronic administration of RO5256390 (RO-90) on the excitability of dopamine neurons of the ventral tegmental area (VTA). Up: representative recordings from dopamine neurons from a vehicle- (left) and RO5256390-treated rat (right); bottom: summary effect from 61 neurons from 5 vehicle- and 122 neurons from 5 RO5256390-treated rats; AP: action potential (spike); ISI: interspike interval; ***p < 0.001, two-tailed Student’s *t*-test.

Chronic RO5256390 led to a significant (*p* < 0.001, two-tailed Student’s *t*-test, data from 85 neurons from 5 vehicle- and 122 neurons from 5 RO5256390-treated rats) increase in the firing rate of dopamine neurons, frequency of burst-like firing, percent of spikes which occurred within the burst, and mean number of spikes in burst. Other characteristics of excitability of dopamine neurons were not affected by RO5256390 (Fig. 6; the characteristics of the neuronal excitability in RO5256390-treated rats are shown as %±SEM of the corresponding characteristics in the vehicle-treated controls to allow the presentation of these characteristics at the same graph, statistical assessment was performed on the actual, non-normalized values).

## Discussion

Is this study we examined, for the first time, the effect of an agonist (RO5256390) and a partial agonist (RO5263397) of TAAR1 on in vivo excitability of 5-HT neurons of the DRN, noradrenaline neurons of the LC, and dopamine neurons of the VTA. We found that acute administration of RO5256390 inhibited 5-HT neurons of the DRN and dopamine neurons of the VTA. This effect of RO5256390 was reversed by the subsequent administration of RO5263397 and prevented by the earlier administration of RO5263397. Chronic administration of RO5256390 led, however, to increased excitability of 5-HT neurons of the DRN and dopamine neurons of the VTA. With regards to 5-HT neurons, chronic RO5256390 increased the frequency of their burst-like activity. With regards to dopamine neurons, chronic RO5256390 boosted their firing rate as well as burst activity.

The excitability characteristics of catecholamine neurons in control animals were similar to those observed in our previous studies (Table 1) [13, 15, 16]. Even though the firing activity of 5-HT neurons of the DRN was higher than that observed in Sprague–Dawley rats [13], it was in the similar range as measured in our previous experiments in Wistar rats [14–17].

We found that acute administration of RO5256390 significantly and dose-dependently inhibited 5-HT neurons of the DRN (Fig. 1). This result is consistent with previous studies, which reported inhibitory effect of acute administration of RO5256390 [7] and of another agonist of TAAR1, RO5166017 [9], on the excitability of 5-HT neurons in brain slices. We also found that RO5256390-induced inhibition of 5-HT neurons was reversed, in a dose-dependent manner, by the subsequent administration of RO5263397. It is thus likely that the effect of RO5256390 is indeed mediated via TAAR1 receptors, putatively, TAAR1 receptors expressed in the DRN [8]. However, since the firing of 5-HT neurons, after its recovery by RO5263397, was re-inhibited by the subsequent administration of 8-OH-DPAT, 5-HT_1A_ receptors might be involved in the modulation of excitability of 5-HT neurons by TAAR1 ligands.

In the present study, acute administration of RO5256390 and RO5263397 did not alter the firing activity of noradrenaline neurons of the LC. This finding is not surprising as TAAR1 receptors were found to be absent in the LC while they were present in the DRN and VTA [8].

We found that acute RO5263397 did not alter the excitability of 5-HT neurons of DRN in in vivo conditions, neither did RO5256390, which was administered subsequently. Interestingly, RO5263397 suppressed the burst firing of 5-HT neurons. The final administration of WAY100135 tended to stimulate the firing of 5-HT neurons, but this effect was not statistically significant (Fig. 2). The results of the present study suggest that the blockade of TAAR1 receptors by RO5263397 does not alter the excitability of 5-HT neurons in in vivo conditions, but it prevents their subsequent activation by RO5256390. It was however reported in a previous study that RO5263397 stimulated the firing activity of 5-HT neurons in brain slices [7]. It is possible that in ex vivo conditions 5-HT neurons are directly stimulated by the blockade of TAAR1 receptors expressed in the DRN. In in vivo conditions, RO5263397-mediated blockade of TAAR1 receptors in another brain area (e.g., PFC) putatively neutralize the direct effect of RO5263397 on TAAR1 receptors expressed in the DRN. With regards to the lack of statistically significant effect of WAY100135, administered after RO5263397 and RO5263397, on the firing activity of 5-HT neurons, it might be due to functional interactions between TAAR1 and 5-HT_1A_ receptors. The pleiotropy between TAAR1 and 5-HT_1A_ receptors was indeed suggested by De Gregorio and colleagues [26].

Similar to its inhibitory effect on 5-HT neurons of the DRN, acute RO5256390 significantly and dose-dependently inhibited dopamine neurons of the VTA as well (Fig. 3). Acute RO5256390 also supressed the burst firing of dopamine neurons. This is consistent with the inhibitory effect of RO5256390 on dopamine neurons, previously observed in brain slices [7]. As in the case with 5-HT neurons of the DRN, RO5256390-induced inhibition of dopamine neurons of the VTA was reversed by RO5263397, in a dose-dependent manner. The recovery of dopamine neuronal firing activity after RO5263397 was however only partial; the complete re-activation of dopamine neurons was observed only after the administration of haloperidol. This combined effect of RO5263397 and haloperidol might be due to the functional interactions between TAAR1 and D_2_ receptors, described in previous publications [7, 26–29].

As in the case with 5-HT neurons of the DRN, RO5263397 did not alter the excitability of dopamine neurons in in vivo conditions but prevented their inhibition by the subsequent administration of RO5256390 (Fig. 4). A previous study, however, reported that RO5263397 stimulated dopamine neurons of the VTA in in vivo conditions. Similarly to what was said above regarding 5-HT neurons of the DRN, it is possible that in ex vivo conditions the blockade of VTA TAAR1 receptors by TAAR1 led the activation of dopamine neurons. In an intact brain, targeting the TAAR1 receptors in another brain area (e.g., NAcc) might diminish the effect of RO5263397 on TAAR1 expressed in the VTA. It was previously reported that acute administration of another antagonist of TAAR1, EPPTB, stimulated dopamine neurons of the VTA in in vivo conditions [10]. It is thus possible that the functional affinity of EPPTB to the TAAR1 receptors regulating the excitability of dopamine neurons in in vivo conditions is higher than that of RO5263397.

To the authors’ best knowledge, our study is the first one to investigate the effects of chronic administration of the selective TAAR1 agonist RO5256390 on the excitability of 5-HT neurons of the DRN and dopamine neurons of the VTA. With regards to 5-HT neurons of the DRN, their inhibition, observed after acute RO5256390, disappears after chronic administration of this ligand. Furthermore, 5-HT neurons of rats chronically treated with agonist RO5256390 exhibited higher frequency of the burst-like firing, comparing to the vehicle-treated controls (Fig. 5).

The incidence of the acute inhibitory effect on 5-HT neuronal firing activity, which is disappearing after chronic treatment with the same drug, is not unique for RO5256390. It was also reported for multiple antidepressant drugs, such as selective 5-HT (SSRIs) [30] and dual 5-HT and noradrenaline (SNRIs) reuptake inhibitors [31–33]. The acute administration of the atypical antipsychotic drug aripiprazole decreased the firing rate of 5-HT neurons of the DRN, however, chronic treatment with the same drug increased the mean firing rate of 5-HT neurons [34]. The mechanism underlying the acute inhibitory effect of acute SSRIs, SNRIs, and some atypical antipsychotics is the elevation of extracellular 5-HT levels and activation of 5-HT_1A_ autoreceptors. Chronic SSRI, SNRI or aripiprazole treatments led to desensitization of 5-HT_1A_ autoreceptors, resulting in the restoration of the normal or even in the increase of firing activity of 5-HT neurons [35, 36]. Since transgenic mice overexpressing TAAR1 were shown to have increased extracellular 5-HT levels [8], it is likely that the activation of 5-HT_1A_ autoreceptors is responsible for the acute RO5256390-induced inhibition of 5-HT neurons. The short-loop (involving 5-HT_1A_ autoreceptors expressed on the cell bodies of 5-HT neurons of the DRN), as well as long-loop (involving 5-HT_1A_ heteroreceptors expressed on DRN-projecting pyramidal neurons of the PFC) [37] inhibitory circuits might be involved in the acute RO5256390-induced suppression of 5-HT neurons. The finding that RO5256390 failed to suppress 5-HT neuronal firing activity in rats pre-treated with PCPA which inhibits 5-HT synthesis supports this suggestion. It is also possible that chronic RO5256390 administration leads to desensitization of 5-HT_1A_ receptors. Further studies are however required to test this hypothesis.

To the authors’ best knowledge, RO5256390 is so far the only drug amending the internal pattern of the action potentials generation in 5-HT neurons, without altering their mean firing rate. It is thus likely that TAAR1 receptors play a role in the modulation of the excitability pattern of 5-HT neurons. It was previously reported that in 5-HT neurons, the burst-like mode of firing enhances both the release of 5-HT and its postsynaptic effect, in comparison with the same amount of action potentials fired in a single-spike mode [38]. The present finding of the stimulatory effect of chronic RO5256390 on the burst firing of 5-HT neurons is therefore consistent with the observation of increased PFC 5-HT levels in mice overexpressing TAAR1 [8]. TAAR1 agonists, after their sustained administration, might therefore increase the efficiency of 5-HT neurotransmission by the modulation of the architecture of firing of 5-HT neurons. As such, they have the potential to be a new type of CNS drugs.

We found that while acute RO5256390 supressed dopamine neurons of the VTA, chronic administration of this TAAR1 agonist robustly increased the mean firing rate of dopamine neurons of the VTA (Fig. 6). Since acute TAAR1 overexpression resulted in increased extracellular dopamine levels [8], it is possible that the activation of D_2_ receptors is involved in RO5256390-induced inhibition of dopamine neurons. Desensitization of D_2_ receptors might thus be involved in the switch from the inhibition of dopamine neurons after the acute treatment to their activation after the chronic treatment with RO5256390. This hypothesis should be explicitly elucidated in future studies.

We found that chronic RO5256390 not only increased the mean spontaneous firing rate of dopamine neurons of the VTA, but also their burst-like mode of firing. As in the case with 5-HT neurons, the burst-like mode of firing of dopamine neurons boosts the nerve terminal transmitter release, in comparison with the same amount of action potentials fired in a single-spike mode [39]. It is thus likely that the sustained activation of TAAR1 receptors stimulates central dopamine neurotransmission via the increase of firing rate of mesolimbic dopamine neurons, as well as via the triggering of their burst-like activity. This suggestion is consistent with the finding of increased NAcc dopamine levels in mice overexpressing TAAR1 [8].

The fact that the effects of the TAAR1 ligands were examined in male rats only is a limitation of the present study. The possible sex differences in the TAAR1-mediated modulation of monoamine neuronal firing activity should be examined in future studies.

In summary, acute administration of TAAR1 agonists leads to the inhibition of 5-HT neurons of the DRN and dopamine neurons of the VTA, *via* the mechanisms putatively involving activation of 5-HT_1A_ and D_2_ autoreceptors, respectively. Compounds with TAAR1 antagonistic property might not alter the excitability of 5-HT and dopamine neurons in in vivo conditions, but they prevent or reverse the abovementioned effect of TAAR1 agonists. The inhibition of 5-HT and dopamine neurons disappear after chronic activation of TAAR1 receptors, *via* a mechanism putatively involving desensitization of 5-HT_1A_ and D_2_ autoreceptors. Furthermore, sustained activation of TAAR1 receptors increases the firing rate of dopamine neurons and stimulates bust-like mode of firing of 5-HT and dopamine neurons. Boosting of 5-HT and dopamine neurotransmission *via* the modulation of 5-HT and dopamine neurons might be involved in antidepressant- and antipsychotic-like behavioral effects of TAAR1 ligands, reported by Revel et al. [7]. This hypothesis should be tested in future studies using animal models of depression and schizophrenia.

## Supplementary information

Supplemental material

## Animal experiments

All experimental procedures were approved by the Animal Health and Animal Welfare Division of the State Veterinary and Food Administration of the Slovak Republic (Permit number Ro 3592/15-221) and confirmed to the Directive 2010/63/EU of the European Parliament and of the Council on the Protection of Animals Used for Scientific Purposes.
