# Supplementary material for: Effects of acute and chronic administration of trace amine-associated receptor 1 (TAAR1) ligands on in vivo excitability of central monoamine-secreting neurons in rats
Source: Mol Psychiatry. 2022 Sep 1;27(12):4861–8. doi: 10.1038/s41380-022-01739-9 (PMC9763099; doi:10.1038/s41380-022-01739-9)
Supplement: Supplementary file 1 — Supplemental material [file 41380_2022_1739_MOESM1_ESM.pdf]

Effects of acute and chronic administration of trace amine-associated receptor 1 (TAAR1) ligands on in vivo excitability of central monoamine-secreting neurons in rats.

Daniil Grinchii, Marius C. Hoener, Talah Khoury, Roman Dekhtiarenko, Reyhaneh Nejati Bervanlou, Daniela Jezova, and Eliyahu Dremencov.

Supplementary materials

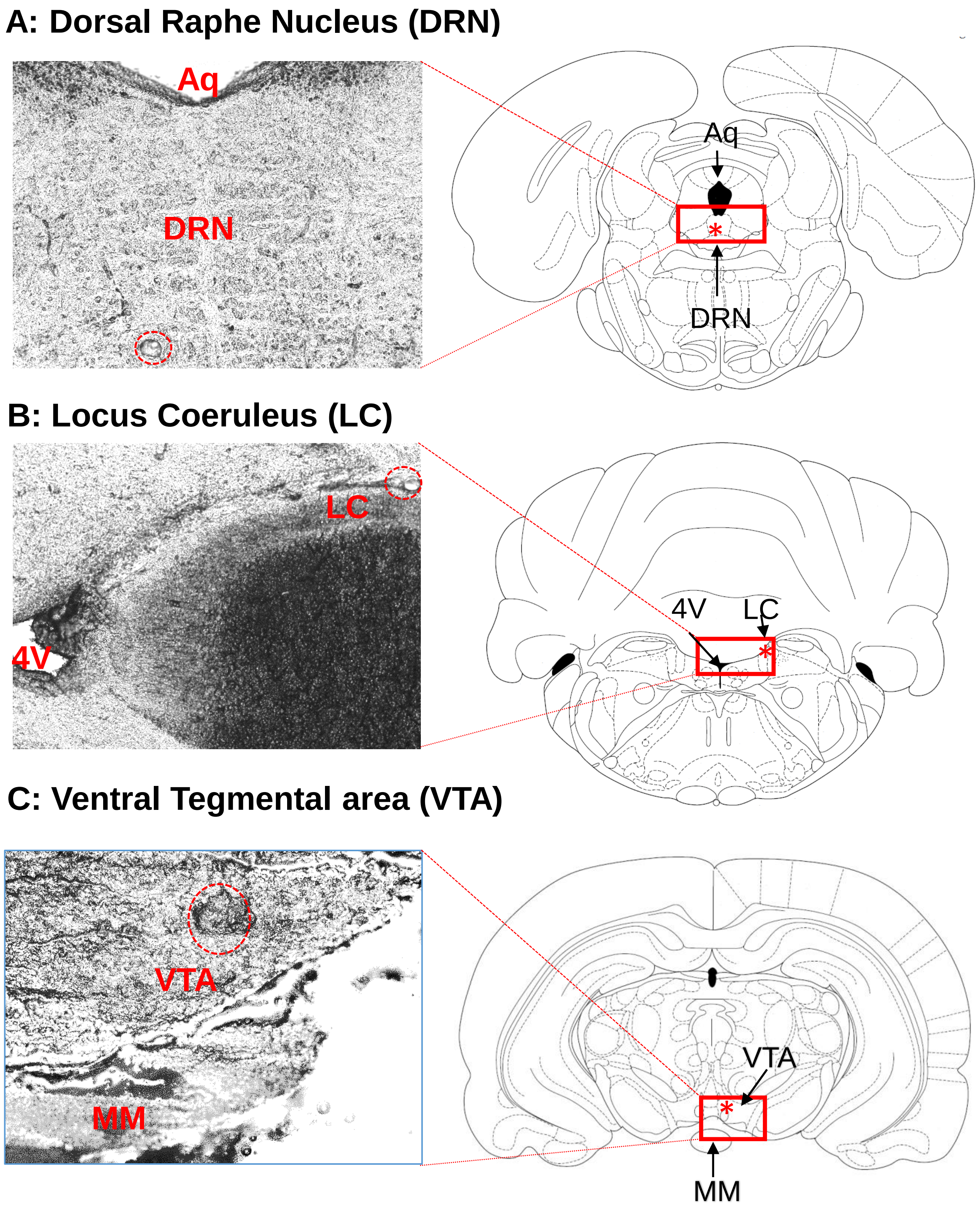

Figure S1: Verification of the electrode tip location within the DRN, LC, and DRN. After completion of electrophysiological recordings, the animals were euthanized by overdose of chloral hydrate. In selected animals, the electrode tip location was labeled by electrolytic lesion using a direct current (DC) of 0.5 mA for 15 s. Rats' brains were removed and fixed in 10% paraformaldehyde for 24 h, and afterward in 30% sucrose for 7 days. Frozen sections were cut at 50 mm and examined under a light microscope to verify the placement of the electrode tip in the DRN (A), LC (B), or VTA (C). Aq: cerebral aqueduct; 4V; fourth cerebral ventriculi; MM: medial mammillary nuclei; / * :electrode tip location/lesion.

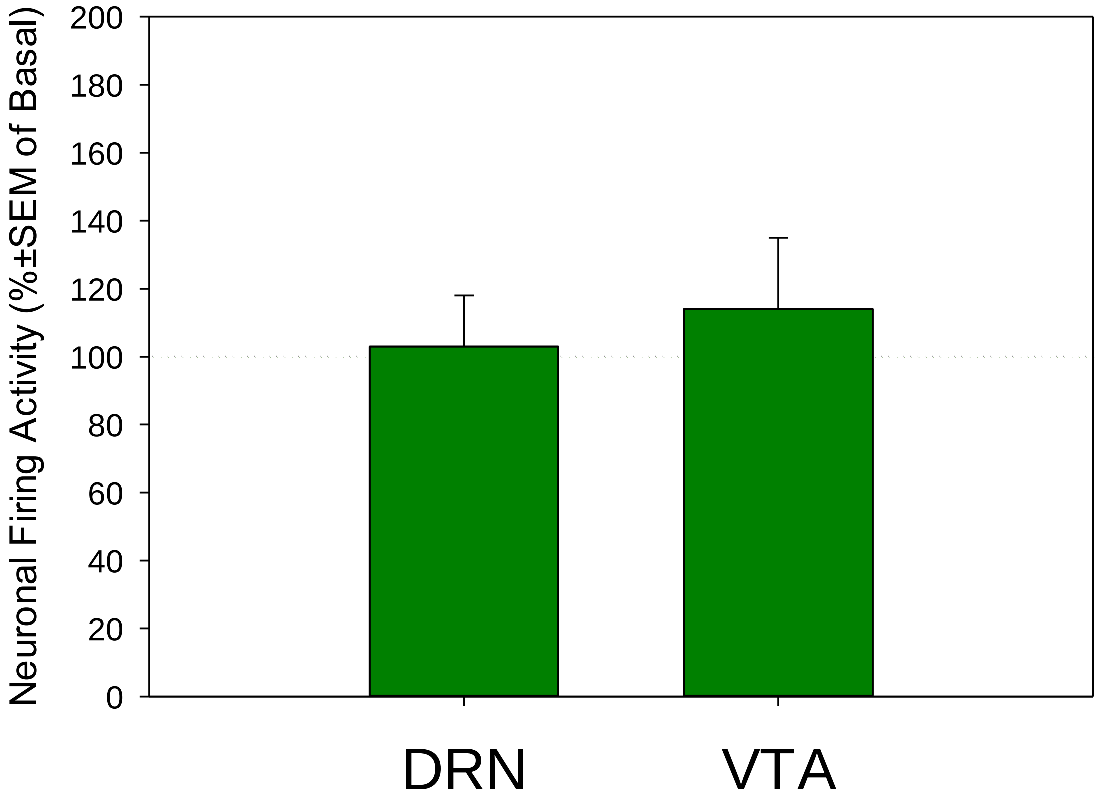

Figure S2: Lack of effect of the vehicle solution on the firing activity of serotonin (5-HT) neurons of the dorsal raphe nucleus (DRN) and dopamine neurons of the ventral tegmental area (VTA). After after a DRN 5-HT or VTA dopamine neuron was identified and its basal firing activity was recorded for 2 min, vehicle (0.3% polysorbate-80 in 0.9% sodium chloride; 3 ml/kg) was administered *via* a catheter placed in a femoral vein. The firing activities of 5-HT and dopamine neurons after vehicle administration are expressed as %±SEM of their basal firing activities (taken as 100% and shown by the dotted line). Vehicle did not alter the firing activity of 5-HT and dopamine neurons in a statistically significant way.

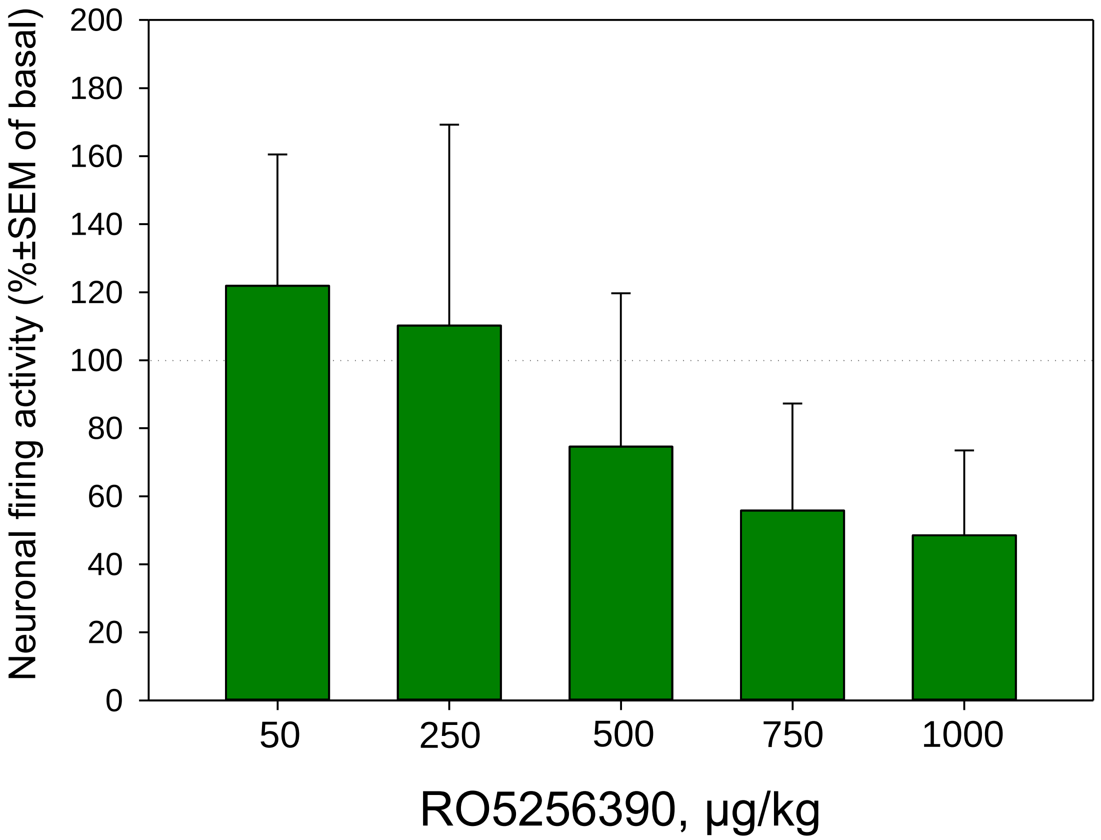

Figure S3: Lack of effect of the full agonist trace amine associated receptor 1 (TAAR1) RO5256390 on the firing activity of serotonin (5-HT) neurons of the dorsal raphe nucleus (DRN) in rats pre-treated with p-chlorophenylalanine (PCPA). Rats were pre-treated with the 5-HT synthesis inhibitor PCPA (300 mg/kg) for three days before electrophysiological experiments. After after a neuron was identified and its basal firing activity was recorded for 2 min, RO5256390 (50-1000 µg/kg) was administered *via* a catheter placed in a femoral vein. The firing activity of 5-HT neurons after the administration of each dose of RO5256390 is expressed as %±SEM of the basal firing activity (taken as 100% and shown by the dotted line). RO5256390 did not alter the firing activity of 5-HT neurons on PCPA-pre-treated rats in a statistically significant way.

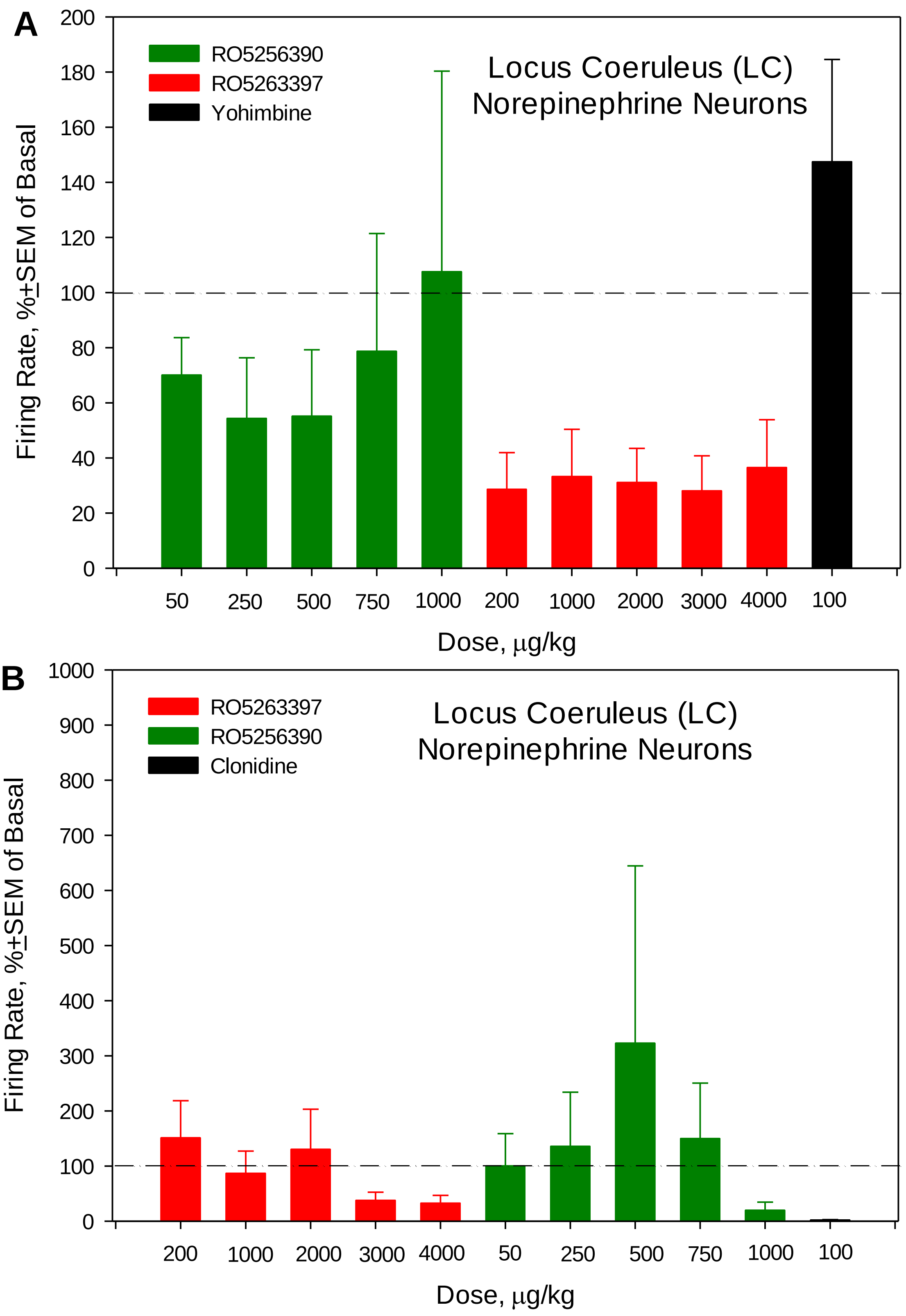

Figure S4: Effects of the full (RO5256390) and partial (RO5263397) agonists of trace amine associated receptor 1 (TAAR1) and of antagonist (yohimbine) and agonist (clonidine) of α_2-adrenoceptors on the firing activity of noradrenaline neurons of the locus coeruleus (LC). A: RO5256390 administered first, followed by RO5263397 and yohimbine; B: RO5263397 administered first, followed by RO5256390 and clonidine. The firing activity of noardrenaline neurons is expressed as %±SEM of the basal firing activity (taken as 100% and shown by the dotted line). RO5256390 and RO5263397 did not alter the firing activity of noradrenaline neurons in a statistically significant way, regardless the order of their administration.

**Characteristic**	**DRN**	**LC**	**VTA**
**RO5256390, 1 mg/kg:**			
Frequency of the bursts	288 ± 182	111 ± 61	32 ± 17**
Fraction of the action potentials occurring in bursts	434 ± 211	111 ± 44	68 ± 23
Mean number of the action potentials in burst	80 ± 20	126 ± 33	104 ± 14
Mean interspike interval, ms	62 ±18	99 ± 10	116 ±36
**RO5263397, 4 mg/kg**			
Frequency of the bursts	29 ± 12**	30 ± 20	131 ± 70
Fraction of the action potentials occurring in bursts	41 ± 19*	81 ± 24	149 ± 83
Mean number of the action potentials in burst	76 ± 26	78 ± 10	141 ± 24
Mean interspike interval, ms	75 ± 25	87 ± 5	137 ± 32

Table S1: Effect of acute RO5256390 and RO5263397 on the burst activity of serotonin (5-HT) neurons of the dorsal raphe nucleus (DRN), noradrenaline neurons of the locus coeruleus (LC), and dopamine neurons of the ventral tegmental area (VTA). *p<0.05 and **p<0.01 in comparison with baseline, Bonferroni post-hoc test.
